# Book Reviews

**Published:** 1820-02

**Authors:** 


### CRITICAL ANALYSIS

OF

### RECENT PUBLICATIONS, IN THE DIFFERENT BRANCHES OF MEDICINE AND SURGERY.

"I would have men know, that, though I reprehend the easie passing over of the causes of things, "by ascribing them to secret and hidden vertues and properties; (for this hath arrested and laid "asleepe all true enquiry and indications;) yet I doe not understand but that, in the practical "part of knowledge, much will be left to experience and probation, whereunto indication cannot "so fully reach: and this not only in *specie*, but in *individuo*. Yet it was well said, *Vere scire* "*esse per causas scire*."—BACON.

*Essays on Hydrocephalus Acutus, or Water in the Brain.* By J. CHEYNE, M.D. F.R.S.E. &c. Second Edition. 8vo. pp. 168. Hodges and M'Arthur, Dublin; and Longman and Co. London. 1819.

ELEVEN years have elapsed since Dr. Cheyne published his first Essay on Hydrocephalus, during which period his experience has tended to confirm his sentiments respecting the nature and treatment of that disease. It is not customary in us to take up the second edition of a work that has been so long before the public; but, since no account of the former was given in this Journal, and since the author has added some original matter to the present, we think proper to take a view of his observations and opinions on the present occasion.

In order to show what were the opinions respecting acute hydrocephalus prevalent in England at that period, and his own claims to originality of views respecting its origin, Dr. Cheyne prefaced his first Essay with a historical account of the Treatises which had been previously published on this subject in the English language; commencing, in the year 1733, with Dr. St. CLAIR, or rather Mr. JOHN PAISLEY, of Glasgow; and ending with the Memoir of Dr. GARNET, published in this Journal in 1801: he has not now extended this to later writers, because "the animadversion of a fellow-candidate for professional approbation might appear invidious." The greater part of the works of later English authors have been noticed in this Journal, and several good memoirs and observations have been published in it; amongst which we may particularly point out those of Mr. SPRY, (vol. ii.) Dr. CLUTTERBUCK, (*ibid.*) Mr. REEVE, (vol. iii.) Mr. WHITE, of Bath, (*ibid.*) Mr. SHAW, of Dudley, (*ibid.*) Mr. J. B. DAVIS, (vol. viii.) Mr. BARTLETT, (vol. xi.) Dr. PATTERSON, (vol. xv.) Dr. COXE, (vol. xviii.) Dr. FOTHERGILL, (*ibid.*) Dr. ROBERTON, (vol. xxi. xxii. xxiii. xxiv.) Dr. YEATS, (vol. xxxiv. xxxv. and xxxvii.) and Dr. REGNAULT, (vol. xli.) We shall take occasion to refer to some other writers, when any remarks likely to be of utility may be elicited in our progress through the work of Dr. Cheyne.*

"Hydrocephalus acutus," Dr. Cheyne commences with observing, "occurs chiefly in childhood. It is attended with pyrexia, and with symptoms indicative of a diseased state of the sensorium. It generally terminates in death; after which, along with other morbid appearances, the ventricles of the brain are almost always found enlarged, and full of lymph." Like all general definitions of disease, this, as the author elsewhere points out, will be found far from being accurately applicable to many particular cases; since the affection sometimes terminates fatally without any serous effusion in the brain having taken place; and, in some few instances, when such an effusion to a considerable extent has been witnessed, the intellectual faculties have remained unaffected to the last moment of the patient's existence. It is more easy to give precise descriptions of the grand characteristics of its most common

---

* As this is so interesting a subject, and one on which information derived from extensive sources, under different circumstances of climate and the constitution and mode of education of its subjects, is of so much importance, we shall give the titles of the best works and essays, besides those above mentioned, which have been written on it, that are not noticed by Dr. Cheyne; and to which we shall refer in the course of our review of his essays. These references may be of utility to the more erudite and studious part of our readers.

Vesalius, *Opera*, lib. i. c. 5.

Ludwig, *Dissertatio de Hydrope Cerebri Puerorum;* 1774.

Odier, in the *Memoires de la Societé Royale de Medecine*, année 1779, p. 198; and in his *Manuel de Medecine Pratique*, Geneve, 1803.

Bursérius, in his *Institutiones Medic. Pract.* tom. iii. p. 50.

Camper, *Mem. de la Societé Royale de Medecine*, année 1785, p. 60.

Wahn, in the *Journal de Medecine Chir. et Pharm.* 1791.

Weikard, *Medicinischen Fragmenten und Erinnerungen*, seit. 112-119, 1791.

Hufeland, in his *Handbüchern über Kinderkrankheiten*, 1798, Berlin.

Rowley, *A Treatise on the new-discovered Dropsy of the Membranes of the Brain*, 1801, London.

Hopfengartner, *Untersuchung über die Natur und Behandlung der Verschiedenen Arten der Gehirnwassersucht*, 1802, Stuttgart.

Wenzels, J. und C., *Bemerkungen über die Hirnwassersucht*, 1806, Tübingen.

Jadelot, in the *Journal de Med. Chir. et Pharm.* 1806, Paris.

Formey, *Uber die Wassersucht der Gehirnhohlen*, 1810, Berlin.

Boyer, in his *Traité des Maladies Chirurgicales*, tome v.

Portenschlag-Ledermayer, J. E. von, *Uber den Wasserkopf*, 1812, Wien.

Thomson, A. T., in the *London Medical Repository*, vol. i. 1813.

Goelis, in his *Praktische Abhandlungen über die vorzüglicheren Krankheiten des Kindlichen Alters, Erster Band. Von der Hitzigen Gehirnhöhlen-Wassersucht*, 1815, Wien. *Uber den Chronischen Wasserkopf*, 1818, Wien.

Yeats, *A Statement of the Early Symptoms of Water in the Brain*, &c. 1815; and *an Appendix to the Pamphlet on the Early Symptoms of Water in the Brain*, 1819. London, Burgess and Hill.

Coindet, *Memoire sur l'Hydrocephale, ou Cephalite interne Hydrocephalique*, 1817. Paris et Geneve.

Porter, in the *Quarterly Medico-Chirurgical Journal*, No. iii.

Brachet, *Essai sur l'Hydrocephalite aiguë des Ventricles du Cerveau*, 1819. Paris.

forms: these, Dr. Cheyne considers, evidently indicate a difference in the circumstances favouring their origin.

"In the first," he says, "we find, before any characteristic signs, that the patient for some days, or even weeks, has complained of pains in his head or belly; while at the same time he has been slightly feverish, dull, vertiginous, sallow, without appetite, or perhaps with an increased or capricious appetite, and with a considerable disorder in all the functions of the abdominal viscera. In some instances a dragging of one of the legs has been observed, which has led to a fruitless examination of the hip-joint and spine; in others, a very painful crick in the neck has taken place, as the first symptom of disease. These complaints arise gradually, and the child's friends are not awakened to a sense of danger, until, advancing a step farther, the commencement of a specific disease has more distinctly shown itself. The dullness and severe pains in the head are now accompanied with vomiting, usually upon getting up in the morning, or after the child has begun to stir about. Yet even this symptom is often disregarded until the second or third day of its recurrence, and the disease has made considerable progress before the illness of the patient is suspected to arise from a disordered condition of the brain.

"When the attention is more particularly excited by these symptoms, the head-ach, or pain in the forehead, will be observed returning at shorter intervals. The child often affectingly complains of his head; he sighs frequently, is dull; his head requires to be supported; he complains of weariness in his eyes; the pupils appear unusually contracted, and he has an aversion to light, but in the dark he sometimes fancies he sees flashes of light. His tongue is white, and his belly generally costive. The stools, at first, are of the colour of clay; but, as the disease advances, they become of a gelatinous consistence, of a dark-green colour, and they have a peculiar smell, not unlike the smell of the breath in the beginning of some of the exanthemata. The pulse becomes quick; and at particular times these symptoms are attended with febrile heat and irritability, and the child complains not of head-ach only, but of pains in different parts of the body, which are sometimes exceedingly acute. At one time he will complain of pains in his limbs; at another, of pain in his breast, or in the nape of the neck, very often in his bowels; but, before his friends can make any preparation to relieve him, the pain ceases, or is transferred to some other part: at another time he will lie long on his mother's knee, restless and whining, from dull rheumatic pains. These disorders cannot last long without impairing the child's strength; and, accordingly, in ten days or a fortnight, the period usually occupied by the first stage of this attack, his appearance is altered, his manner becomes peevish, his hand tremulous, and his gait tottering.

"II.—In the second form of the attack, which is less frequent, the disease runs a more rapid course. After the child has been in a drooping state for a short time, which, although it sometimes may escape observation, is generally recollected, there is a sudden change to a fever, attended, even from the first, with a great degree of pyrexia, with frequent but short and irregular remissions, flushing, severe head-ach, tenderness all over the abdomen, and increased sensibility, and sometimes brilliancy, of the eyes. It is said to be often difficult immediately to distinguish hydrocephalus from fever, and this is the form in which there is the greatest resemblance between the two diseases; but we are led to suspect some deeply-seated evil, from his frantic screams and complaints of his head and belly, alternating with stupor, or rather lowness and unwillingness to be roused; and we are struck with the great irritability of the stomach, which exists in a degree beyond what we generally find it in the fevers of this country,—retching and vomiting being brought on by a change of posture, and certainly by every attempt to sit up in bed; and the disordered state of the bowels which attends this irritability of the stomach, is also remarkable. And, when at any time the child has a little respite from the violence of these symptoms, we find our suspicions confirmed by his look; for, when the features do not express pain or terror, there is not unfrequently a vacancy of look,—the eyes being set, with an expression of dejection, which is peculiar to certain diseases of the brain.

"III.—I have observed, less frequently than the first case, yet I think more so than the second, what may be described as the third form of hydrocephalus. This last may be considered as an instance of conversion of disease, and it is attended with considerable variety of symptoms. When hydrocephalus arises after an indifferent state of health, as, for example, after there had existed a scrofulous disease which has subsided; or where, from predisposition, and from the anomalism of the symptoms, some such disease might have been expected; or where the child has had some epidemic disease, as measles or scarlatina, from which he has not perfectly recovered or regained sound health,—the attack is sometimes made with all the violence which I have described as distinguishing the second form. When again the attack comes on as the sequel of an acute disease, as, for instance, remittent fever, hooping-cough, dentition, the child almost imperceptibly slips into hydrocephalus; there are scarcely any of the symptoms of the early stages; and palsy and convulsions are sometimes the first indications of the new disorder. In such instances, however, were we not lulled into security by the previous disease, we might perhaps sooner detect a tendency to hydrocephalus. I have reason to think, in one case of this variety, that the symptoms resembled those described in the first form of the attack, but they were thought a part of the original disease. Instances of hydrocephalus unattended with pain, are mentioned by more than one author, and have repeatedly occurred in my practice. Dr. Quin adverts to a case, in which, during the child's illness, there was no symptom to suggest the real cause of death, yet, when the body was examined, a large quantity of fluid was found within the ventricles of the brain.

"The situation of the patient is not yet desperate; for, after the appearance of most, or even of all, the symptoms which have been enumerated in describing the first two forms of hydrocephalus, the child sometimes recovers. Cases are recorded, in which, without the use of any active medicine, the disease has gradually subsided, leaving the child in the enjoyment of health."

We have transcribed these descriptions fully, because they are already as concise as is consistent with perspicuity. The first form, Dr. Cheyne considers as the most favourable; though, perhaps, the second might be equally so, were it detected at an early period: the first two or three days are, in the latter, generally decisive, and these are frequently lost. This form is the most regular in its progress, the three stages observed by Dr. Whytt being generally distinctly marked. The foregoing descriptions are, however, only applicable to the commencement of the disease: as it proceeds, there is but little diversity in its symptoms, and in all its stages and forms there are signs of irregular excitement; becoming, however, more evident as the disease becomes more fully established. The heat of the body is increased, the skin is very dry; there is a deep blush on the face, or on one cheek only; and the breathing is sighing, laborious, and quick. At another time, the blood circulates more equally; the skin is of a natural warmth, or moist with perspiration; the countenance is pale; and the breathing so gentle that it cannot be heard. The appetite and thirst vary; sometimes exhibiting nearly a natural state of the stomach: at other times, the fetid breath which has erroneously been supposed peculiar to this disease, the total loss of appetite, and a continued vomiting, even for days, show the stomach to be in the greatest disorder. The bowels are never regular: they are generally slow, but now and then a severe bilious purging attends the vomiting. The urine is sometimes retained for twenty-four or thirty-six hours; at other times there is a frequent, or even constant, desire to pass it, attended with great pain. The senses are often entire, or perhaps morbidly acute; the retina, in the early stage, being generally very sensible to light, and the ear to sound; sometimes the intellects are deranged from the onset, and there is great dullness of sensual perception; and, in some instances, after delirium, convulsions, and insensibility to external objects, a complete return of the intellects has been observed to occur shortly before death. This is an abstract of the author's delineation of the more general characters of the disease.

The three stages described by Whytt, it is remarked, may sometimes be found designated with tolerable precision. Goelis, however, makes the disease to consist of four stages, by dividing the first, or inflammatory stage, of Whytt, into two: one, a state of preternatural turgescence of the cerebral vessels; the other, that of actual inflammation, occurring in the order above described. He thinks, careful observation will often lead to a discovery of various symptoms indicative of the approach of the inflammatory stage, some time previously to its actual presence. The most strongly-marked presages, he considers, are to be derived from—

1°. *The manifestations of the mental faculties.*—A change of the natural cheerfulness to a dull, peevish disposition, for some time previously to the appearance of the signs of inflammation, has been remarked as a frequent precursory sign by Whytt, Dr. Cheyne, Goelis, Formey, Hopfengartner, and almost every other author. Portenschlag says, "A diminution of the natural activity, and especially of the prattling talk, is hardly ever an illusive sign." It has often been remarked, too, after Paisley, that the child has been more active and cheerful in the morning than in the evening; a dull head-ach coming on at the latter period, and leading to a restless night. Pain in the head is sometimes absent in this state; and Dr. Yeats thinks that the disease may be making considerable progress before it is complained of; but it is much more frequently present, and sometimes for a very considerable period. The Wenzels and Borelli have seen it continue during four months previously to the clear development of the inflammatory stage of the disease.

2°. *From the functions of the organs of the senses.*—The sight is often dull and imperfect. Formey, as well as Goelis, has remarked a sudden loss of speech, on the child's attempting to cry aloud; and this is accompanied with signs of preternatural determination of blood to the head. The hearing is often very acute, so that the slightest noise will make the child start as if alarmed: sometimes, on the contrary, deafness comes on at an early period.

3°. *From the external appearance of the body.*—An unusual largeness of the head is noticed by almost every author. The countenance loses its natural animated appearance; or, as Ludwig, Whytt, Odier, Hopfengartner, Dr. Cheyne, and M. Coindet, have remarked, it varies much in colour. Sometimes one cheek is very red, and the other pallid; and there are often flushed spots in various places. The eyes are dull, often swimming in tears; sometimes there is a dark streak beneath them: the latter is particularly pointed out by Dr. Yeats. The expression of the features of the countenance is often very much altered.

4°. *From the state of sleep and bodily exertion.*—Disturbed sleep is very commonly present in this state, and is noticed by all authors. Gritting of the teeth very frequently attends it; and Coindet says, that he has known this done with such force as to break off small pieces from them. The gait is tottering, and the child often falls.

5°. *From the vital functions.*—The pulse is very irregular: sometimes intermitting almost at regular periods; at others, varying in an indeterminate manner, and with quick accesses of variation. Thus, as Coindet remarks, "if the minute be divided into four equal parts, we shall find in one of them twenty-five pulsations, as if the rate were a hundred in a minute; whilst in another quarter there will be thirty-five or forty, as if the rate were 140 or 160." This sort of irregularity is particularly noticed by Dr. Cheyne; but he speaks of it expressly during the more evidently inflammatory stage of the disease; or, according to Whytt, in the first stage; which is the second, according to Goelis. There is but little confirmed fever; and what there is present comes on especially in paroxysms towards the evening. Medical practitioners should, for this and other reasons, see children presenting the foregoing train of symptoms at this time of the day.

6°. *The secretions and excretions.*—A costive state of the bowels, and decrease in the quantity of the urine, are frequently observed. There is, as Fothergill remarked, heat about the head and the precordia; and Dr. Cheyne and Dr. Yeats observe, that there will be tenderness found about the region of the liver, on making pressure on that part. The stools are often, too, in a very disordered state. The urine often presents a peculiar milky appearance. Formey describes it as having sometimes the appearance of a mixture of three parts of water and one part of milk. This milky appearance of the urine is also noticed by Odier, and Coindet describes it as *un depôt blanc farineux crétacé:* and Vieusseux, as well as Coindet, has found this changed into a sort of micaceous deposition in the second stage of the disease; which matter the latter believes to be urea.

7°. *From the state of digestion and nutrition.*—The appetite varies, but it is almost always diminished; and the nutrition of the body more so than seems to be conformable to the state of the digestive organs, and the quantity of food taken. Vomiting, occurring frequently, especially when the stomach does not contain offensive matter, is the signal, according to the Wenzels, of the breaking-out of the more inflammatory stage of the disease.

The signs of increased excitement of the cerebral system first present themselves, with an accelerated, but often very irregular, pulse, beating at the rate of 50 or 60 in a minute at one instant, and the next at that of 130 or 140, or even more. When this state of excitement subsides, the pulse becomes slow, and more or less of dullness and torpor ensue: there is imperfect vision, and sometimes total blindness, with strabismus; the pupils are usually much dilated, and insensible to light: but the latter is by no means a constant symptom, not even when serous effusion to a considerable extent is present.

When the second stage of Whytt commences, the pain in the head does not always abate; sometimes it has increased; but when the slow pulse has continued for some time, the pain is less complained of. The child is no longer able to sit up; he lies in a dozing, or comatous, state, which is interrupted by startings and wild expressions of pain, and often by a troublesome cough; still, however, there is often not much incoherence of mind. Sometimes this stage advances with regularity, after the child has been eight or ten days ill, and then, perhaps in three or four days more, the pulse becomes as rapid as ever; and thus it marks the commencement of the third stage, which ushers in a still more hopeless series of symptoms. We must add to this the following forcibly-drawn picture from Dr. Cheyne; and then proceed more rapidly through the rest of the history of the disease.

"Whatever difficulty there may be in ascertaining the disease in its commencement, there is no disease so easily distinguished when far advanced. Indeed, how can we mistake, when we see a child rolling his head on the pillow, or perhaps sawing the air with one hand, while the opposite side is palsied; with a hectic on the cheek, the eye-lids half concealing the pupil, and the eye deprived of its vivacity by the filmy appearance of the cornea, the complete dilatation of one or both of the pupils, and the suffusion of the adnata; drawing long sighs; frequently grinding his teeth; incoherent, or in a state of complete insensibility; with a burning heat of his skin, or a sweat forced from every pore: and all these symptoms alternating with, and at last finished by, stertorous breathing and violent convulsions."

Such a regularity in the progress of hydrocephalus is by no means constant: and it often proceeds in a very different manner. Dr. Cheyne has observed a slow pulse from the beginning; and instances have occurred where the pulse has never fallen below the natural standard, even when the disease has continued for several weeks, and terminated fatally. In some cases, too, the intellects have remained undisturbed throughout the whole of its course.

The duration of the last stage is seldom long, but yet the period is very uncertain. A week is, however, but rarely exceeded; but no symptom, the author says, except the hurried breathing, can with certainty be said to indicate the speedy approach of death. The most common duration of the whole of the stages of this malady, is from two to five, and in some few instances six, weeks.

On dissection, the most usual appearances within the cranium are, venous congestion, signs of inflammation of the arachnoid membrane, appearing in the effusion of fluid in the ventricles and over the hemispheres and at the basis of the brain, false membranes, preternatural adhesions, &c. "The substance of the brain," Dr. Cheyne says, "is generally, but not always, soft and blanched, fimbriated, and particularly soft where it forms the ventricles. The substance of the fornix is often like a soft curd." LIEUTAND (*Obs.* 327), Dr. CLARKE, and M. BRACHET, have stated, that they have found a remarkable induration of one of the hemispheres of the cerebellum and medulla oblongata: this we have only noticed after the more chronic forms of the disease. Tubercles are not unfrequently met with in different parts of this organ. WHYTT found a dense, yellowish-coloured tumor, nearly the size of an egg, situate about the origin of the optic nerves: PETIT (*Mem. de l'Acad. Roy. des Sciences*, 1719, p. 99), and, since him, several other anatomists have observed the pituitary gland to be in a schirrous state. But the latter appearances are not found in the proper and simple form of acute hydrocephalus. The plexus choroides is generally in a morbid state, injected with blood, its veins much dilated, cysts of fluid dispersed about it, and sometimes enveloped with coagulated lymph. In a few cases, purulent matter has been found, especially about the basis of the brain. Pseudo-membranes, from effused coagulable lymph, are very frequently met with in the latter situation. Drs. COINDET and PORTER have particularly noticed organic lesions of the pons Varolii, the corpora olivaria, and the cerebellum; and the former has, in almost all cases, he says, found considerable serous effusion in the spinal canal. It may here be worthy of remark, that paralysis more frequently affects the lower than the upper extremities; and is, indeed, generally confined to the former, in the early stage of the disease.

In relation to the two latter observations, the distribution of the arachnoid, as demonstrated by BICHAT, should be borne in mind. This membrane is commonly supposed merely to envelop the brain: it is not generally known that it is reflected over the dura mater, and prolonged into the vertebral canal, in the same manner. In some cases, no serous effusion is found after death, or but a little more than usual, in the ventricles: but here the arachnoid, and its prolongations lining the ventricles, and the choroid plexus, are highly injected with blood; and sometimes the whole of the substance of the brain shows the presence of an undue quantity of that fluid. Several instances are related of preternatural thickness and firmness of the cranium, and obliteration of the sutures, after the more chronic form of the disease, in the works of RUYSCH, SAVIARD, SANDIFORT, and in the Transactions of the Royal Society of London. "In the abdomen," Dr. Cheyne observes, "I have found the intestines inflamed, and constricted as from spasm, and the surface of the liver of a bright-red colour, abounding in minute vessels, and sometimes extensively adhering to the peritoneum.

In many dissections, I have also found the surface of the liver studded with small white tubercles, not larger than grains of mustard. The glands of the mesentery are often diseased; as is evinced by their enlargement, and the caseous depositions which exist in their substance." He has, also, sometimes found remarkable constrictions and volvuli in the intestinal canal, (which M. Coindet also notices,) the probable consequence of irritation and inverted action of those organs.

The morbid appearances about the liver and mesentery observed by Dr. Cheyne, and the tubercles also which so many anatomists have witnessed in the brain, seem to bear a particular relation only to the origin of the more chronic forms of the disease: but, before we enter on this subject, we must notice some of Dr. Cheyne's remarks on the pathognomonics, diagnosis, and symptomatology, of this malady, and the period of life at which it most frequently occurs.

In children at the breast, Dr. Cheyne says, we do not readily distinguish the attacks of hydrocephalus: we, indeed, see somewhat of its peculiar expression; we observe the child moaning, feverish, watchful, frowning; the expression of pain not violent. The disordered state of the bowels, however, presents some peculiarities: the stools are feculent, glairy, and of a dark-brown, or deep-green, colour; and there is not the "flinging-out of the limbs, and extreme impatience, which children evince in colic or griping-pain." "In no other infantine complaint do we perceive the same knitting of the eye-brows unaccompanied with crying." The head hanging over the nurse's shoulder, and the half-closed eye-lids, are alarming symptoms. When the disease has made further progress, there can be but little difficulty in detecting it from the assemblage of symptoms, although but few distinct pathognomonics can be pointed out. When vomiting is present in the early stage of the disease, we have seen it accompanied with a circumstance, that we think very characteristic of the malady, which is, that it only occurs on the child being agitated, and especially on the head being shaken.

The diseases with which acute hydrocephalus is most likely to be confounded, according to the author, are, common continued fever, and the infantine remittent. From the former, the gradual commencement; the more irregular remissions; the state of the stomach; the nature of the excretions; the great aversion to light; the acute pains about the body; the peculiar pain of the head, (which Darwin said might be referred to the lateral parts of it, especially if it be experienced when the child is lying still,) oftener dull than acute, Dr. Cheyne says, but so overpowering, that it does not admit of the head being raised from the pillow; and, when acute, increased by every throb of the artery, or by any muscular exertion; at intervals, too, and these sometimes regular, it darts through the centre of the brain, and the child is roused with an expression of helpless anguish from the dozing which preceded this acute pain, and into which he instantly relapses, when it is gone: these signs sufficiently distinguish it from fever arising from disordered bowels, and accompanied with merely sympathetic irritation of the brain.

Dr. Cheyne then treats, in succession, of the diagnosis between the disease under consideration and some species of convulsions; a peculiar spasmodic complaint, attended with a crowing inspiration, arising from disorder of the stomach and bowels, and the more slight and temporary affections of the brain, from the latter cause: but we have not space to permit us to dwell on these points: the diagnosis cannot be very difficult to any well-informed practitioner.

Hydrocephalus occurs at every period of life, but it is more especially a disease of children, and of the middle years between weaning and puberty. Dr. Cheyne says, he is inclined to think that there is a foundation for the remark of Ludwig, "Post decimum annum ingruens, plerumque puellas adflixit." This has been repeated by M. Coindet, from statistical calculations. Before the tenth, Dr. Cheyne observes that boys and girls seem alike subject to it: yet Dr. Regnault, whose experience has been very extensive, in his memoir on the subject,* says, he has only seen it in boys. This is very singular: our own observation, however, leads us to think that boys are much more subject to it than girls during childhood. The precosity of the intellectual faculties almost constantly witnessed in the subjects of this malady, must have struck every accurate observer; and we are inclined to believe this is very intimately related to the origin of the disease. The patients of it have generally a very large head; they often show a gradual progress, from unusual sprightliness of manner, to a sort of mental disorder bordering on delirium, or rather insanity, before any fever or other signs of bodily disorder are present. Hopfengartner lays much stress on this point: Portenschlag, however, opposes this notion, because he has so often seen the disease in deaf and dumb children; but, from what we have seen and heard of the conduct of children educated in deaf-and-dumb institutions, we are disposed to think his argument rather favours the side of the question he opposes. Attentive observation of those children shows, we think, that their brain is excited more powerfully than that of children in gene-

* *Journal Universal des Sciences Medicales,* tome ix.

ral, by the exertions they make to overcome the difficulties opposed to their full acquaintance with surrounding objects.

In many cases, it seems to be connected with "those peculiarities of skin," Dr. Cheyne remarks, "complexion, and features, which indicate a great tendency to scrofula. Dr. Percival observes, that 'of twenty-two cases of which he had kept notes, eleven were certainly stumous children, and four were probably so.'" This remark has been made respecting *chronic* hydrocephalus by almost every author, writing from experience, from SAUVAGES to the present time.

We now arrive at Dr. Cheyne's opinions respecting the parts primitively affected in the greater number of cases of hydrocephalus. He says,

"When the disease is forming, there is generally a defect in the function of the liver. It seems to admit of only a scanty and imperfect formation of bile, insufficient to stimulate the intestinal canal, which thus becomes torpid, and is sometimes loaded with fœtid clay-coloured excrement. This state of the canal, in conjunction with the disordered condition of the liver, is perhaps one cause of the derangement of the stomach, which is almost invariably present in the beginning of hydrocephalus; although it is not to be denied, that the vomiting, in the more advanced stages of the disorder, is more naturally explained by supposing, as Dr. Whytt has done, that the stomach sympathizes with the diseased state of the brain.

"When hydrocephalus is established, the stools, although scanty, contain an unusual proportion of bile. Indeed, they appear to be solely a mixture of dark bile with the mucus of the intestines; but the state of the bowels shows that this bile is an imperfect secretion, which, I apprehend, is in a great measure the explanation of the torpor of the intestines,—not, in this stage of the disease, that they are slow from paralysis.

"Vertigo occasions a flow of bile into the stomach, and sickness and vomiting; as may be seen in sickness from swinging, or in sea-sickness, and in sickness from vertigo, originating in organic affections of the brain. Injuries of the brain have been succeeded by abscesses of the liver, &c.; and, in dissections after apoplexy, the liver is often found greatly diseased. On the other hand, there are many instances of apoplexy succeeding to jaundice; and sudden death in gouty persons, has apparently arisen from a metastasis from the liver to the brain. Thus, in many diseases, the direct sympathy between the liver and the brain is obvious. Examples of sympathy between the alimentary canal and the brain, must be in the recollection of every reader.

"I am not prepared to frame rules by which we may decide that the symptoms arise from the morbid sympathy of the brain with a deranged state of the stomach or intestines; or, on the contrary, that they originate in a disordered state of the brain, unconnected with any distant organ. Perhaps future observation may prove, that we shall

**not err when we trace the disease to the organ which first has its functions sensibly disturbed: in a great proportion of cases I have thought that the series of diseased actions commenced in the abdominal viscera."**

The correctness of this opinion is, we think, disputable; for, although there may be sufficient evidence to lead us to suppose that hydrocephalus is sometimes a sympathetic affection of gastric disorder, yet the arguments in favour of its by far the most frequent origin as an idiopathic disease, are very forcible, and, we believe, cannot be refuted. In a very great proportion of cases, it has been preceded, sometimes for several weeks or even months, by signs of cerebral excitement long before gastric disorder has existed: the head is also frequently unusually large, and the forehead prominent, with a want of closure of the fontanelles; there is commonly great precocity of the intellects, and often a wildness of manner, with a sparkling appearance of the eyes, for some time previous to its approach. Younger children will often be observed to vacillate much in walking, and those who have just began to walk alone will lose the power to do so: this state is sometimes accompanied, too, with disturbed sleep. Disorder of the bowels does, in general, soon come on after the occurrence of those symptoms; but this is only what takes place in almost all cases of irritation of the brain; and, on careful examination, it will often be found to bear the characters of a sympathetic affection. The vomiting will chiefly occur in obstinate fits on the child's first waking, or be produced by agitating the child; and it is not so readily excited by substances taken into the stomach, as when it arises from idiopathic disorder. Besides, a great many well-marked cases have lately been published, especially on the continent, which have been cured by blood-letting alone, or at least without any means likely to remove disorder of the gastric viscera; and it can very often be traced to external violence. We may add to these remarks, the efficacy of issues in preventing it in families, when all the former children of the same parents have died of the disease, and in patients who have already shown signs of a disposition to it. All these circumstances are expressly in favour of the idiopathic origin of the disease; and some one or other of them may be observed in by far the majority of cases. It is argued that this disposition may have some relation to the origin of the disease, but that gastric disorder is still the immediate exciting cause. This argument will not hold good in opposition to the evidence of the existence of cerebral excitement for so long a period before that of gastric disorder; and, in many cases, of its running its whole course without such disorder ever occurring, or at least only at intervals, or in the latter stage, as appears from the observations of Odier, Camper, Rush, Goelis, Regnault, Coindet, Brachet, andother experienced practitioners.

The argument, because hydrocephalus is, in its first stage, sometimes removed by purgatives, that it is therefore dependant on gastric disorder, would equally well apply to the skin being the seat of pleurisy, because this affection is removed by a blister.

There can, however, be no doubt but that the most frequent causes of this disease are different under different circumstances of climate, habit of body, and the mode of educating infants and children: a rapid view, therefore, of the opinions entertained on these subjects throughout the greater part of the continent of Europe, to place in opposition with those which have been already mentioned, and which are most prevalent in England, may not be devoid of utility. We shall particularly notice the opinions of Goelis, because, from his office as director of the Institution for the reception and education of poor children at Vienna, he has had extraordinary opportunities for observation; and his talents, also, contribute to render his opinions of much authority. He divides the causes of hydrocephalus into predisposing and exciting; and we shall examine them according to his arrangement, although it is, like all other classifications of predisposing and exciting causes of disease, extremely vague: for instance, a blow on the head is considered as a predisposing cause, whilst the application of cold to the same part is termed an exciting one.

1°. *The age of childhood.*—The origin of the disease is here intimately related to the peculiar development of the vital powers in the head, by which it is so liable to be affected by any exciting causes; the various secretions so frequently found about it, too, have an indirect influence in the production of the disease, especially in their suppression;—those are fluxes from the ears and nose, scrofulous ophthalmia, &c. The great vigour of vital action concerned in the development of the intellectual faculties, is considered by Hopfengartner as a very frequent and powerful predisposing cause. Dentition, and the change of the first set of teeth, are also considered to have much influence in this way, by Fermoy, Goelis, Coindet, and Brachet.

2°. *External violence to the head.*—Hopfengartner, with Rush, considers the injuries resulting from this as a frequent and well-determined series of causes. The former has frequently traced a train of symptoms, often running on for a considerable length of time, persisting from the time of the accident to the breaking-out of the disease. Goelis considers the following as the most frequent. *Injuries during parturition:* of the origin of hydrocephalus from those, he notices several instances; and Stoll and Arantius have made similar observations. Agitation and concussions of the brain, from violent rocking in cradles, blows, leaping on the feet from great heights, falls on the head, extraordinary exertions in skipping, are amongst the other causes of this kind; and Goelis asserts, that he could trace by far the greater proportion of the cases occurring to his observation, to some one or other of them. Odier, Hufeland, Wichmann, Lodemann, De Roose, and many other experienced practitioners, acknowledge an equal influence in similar causes. Fermoy has witnessed some interesting cases of this kind, which show how carefully apparently slight affections of the head from those causes should be attended to. They often continue uninterruptedly for a long time, but with casual periods of increased intensity, though not remarked on superficial observation, and at length break out with all the characters of acute hydrocephalus. We cannot but regret to see the explanation which some persons have given of the mode of agency of violence of this kind: one physician, who has adopted the opinions of Dr. Cheyne, but who has applied them to an extent which he is very far from favouring, says, blows on the head will lay the foundation for hydrencephalus, by a diseased impression on the liver and digestive organs. This doctrine is of very pernicious tendency; because it teaches that, as long as the liver and digestive organs are not affected, we have nothing to fear from a blow of the head; and it tends to divert the practitioner from the proper means for preventing the ill effects of injuries of this kind.

3°. Severe coughs, especially hooping-cough, and the frequent use of emetics, by propelling the blood in an inordinate quantity to the head, are other causes of the kind under consideration, which are occasionally important agents.

Disorder of the functions of the abdominal viscera, and worms in the intestines, are next noticed by Goelis; but these are considered to be much less frequent causes than those previously noticed. The stress that is laid on the former, alone sufficiently indicates his opinion on this subject; and he here agrees with the greater number of continental physicians.

A connate peculiar irritability of the nervous system, is designated by Goelis, Brachet, Coindet, and Girtanner, as not an uncommon predisposing cause.

Scrofula is considered by Goelis, Hufeland, and many of the physicians already named, to be a rather frequent cause of the more chronic form of hydrocephalus; but Coindet thinks that this is not so common as is generally supposed: he says, he has seen a multitude of cases in which no suspicion of a scrofulous disposition could be entertained.

The most frequent exciting causes are said to be, cold applied to the head, especially in newly-born infants, fevers, croup, and other inflammatory diseases. The sudden stopping of discharges and removal of eruptions, measles, and scarlet fever, are frequent exciting causes: Coindet witnessed the influence of the latter to a great extent, during the prevalence of that disorder as an epidemic at Geneva, when it was treated by too free exposure to cold air, &c. Goelis, Coindet, Rush, and Lettsom, speak of it as occurring immediately from apparent metastasis of rheumatic inflammation. Several cases have also been witnessed of its occurrence after, or in connexion with, inflammation of the pleura.

Dr. Cheyne's remarks on the opinions of Rush respecting the nature of this disease, shall now be noticed in a particular manner; as we shall by this means be led to point out, as we believe, some other circumstances of importance respecting its origin. He says,

"Dr. Rush observes, that the disease, in its first stage, is the effect of causes which produce a less degree of that inflammation which constitutes phrenitis.

"And that its second stage is the effect of a less degree of that effusion which produces serous apoplexy; and hence he has been induced to call the former stage phrenicula, from its being a diminutive species or state of phrenitis: for the second stage, he has preferred the name of chronic apoplexy.

"Are we to understand that the same causes, operating less powerfully than when they occasion phrenitis, will produce hydrocephalus? This is not exactly what is expressed, but I rather think it is intended. If not, I am at a loss to know what those causes really are which produce a less degree of that inflammation which constitutes phrenitis. The exciting causes of phrenitis, we all know, are excess in wine, external violence, insolation, indulgence in the more violent passions, or indeed excess in any kind of mental exertion. Now, children are not usually subject to the influence of any of these causes, if we except external violence."

This is but a partial account of the causes of phrenitis: it will originate from all the causes productive of inflammation of the serous membranes in other parts of the body. Besides these, children *are* much exposed to great mental exertion, in the mode in which they are educated in the more civilized nations of Europe, by the great efforts made to procure a precocious development of their intellects: and, we may here repeat, it is chiefly in those who manifest this precocity that the disease is observed. It is most prevalent in the most highly educated nations, and especially in those where children are much in the society of sprightly, intelligent persons: it is very common in France and in England. Camper said, it was unknown in Holland; and Tissort asserted the same with respect to Switzerland. These statements are not quite correct; but it is true that hydrocephalus is there of rare occurrence. We proceed with Dr. Cheyne's remarks,

"From the causes of hydrocephalus, Dr. Rush proceeds to explain the morbid condition of the brain. He affirms, that the two diseases differ only in degree: that hydrocephalus is only a diminutive species of phrenitis; and, in calling the former phrenicula, he is followed by later writers. So far has this view prevailed, that the disease, undergoing another change of name, has been called hydrocephalus phreniticus; and this name has been adopted by some late writers.

"Now, it ought to be recollected, that hydrocephalus scarcely ever affects the adult, and that phrenzy seldom occurs in childhood."

We must not here be confused by mere terms. Although *hydrocephalus* is not commonly said to affect adults, *inflammation of the membranes of the brain, often terminating in serous effusion*, is acknowledged to be a disease they are liable to,—not so frequently as children are to hydrocephalus, certainly; but it should be remembered, that the grand centre of vitality changes, after childhood, to the thoracic and the abdominal organs, in succession. Instead of finding inflammation of the serous membrane of the brain, we find, then, inflammation of those of the thorax and of the abdomen a common disease.

The variation in the character of the symptoms between acute hydrocephalus of children and the phrenitis of adults, is similar to what is observed also between inflammation of the pleura in those subjects. Children have inflammation of the pleura, but it is not of the severe and acute character that the pleuritis of adults presents. Dr. Cheyne next remarks,

"Lastly, it ought to be recollected, that, in hydrocephalus, the centre of the brain is the part chiefly affected: at least, in most cases, we find the effusion on the surface of the brain bearing no proportion to that in the ventricles, and the cortical part of the brain is sound, while the central parts are broken and dissolved; whereas, the effects of phrenitis are most remarkable in the more superficial parts, being sometimes apparently confined to the membranes."

This may be the case, (though we would by no means concede this point if it were of importance, but it is not of the least consequence in the pathology of the disease:) "the centre of the brain," or, as the author afterwards, with more propriety of expression, says, "the ventricles," may be chiefly affected in childhood; but it signifies little, in the pathology of the disease, whether the portion of the arachnoid covering the hemispheres, or that lining the ventricles,* be the part of it chiefly affected. Besides, it is a well-founded doubt whether the greater readiness

---

* See Bichat's demonstration of the distribution of the arachnoid in his *Traité des Membranes*.

with which the brain gives way to distension in children than in adults, may not influence this circumstance; and, we may add, whether death is not the consequence of such effusion sooner in adults than in children. With regard to the brain being "broken and dissolved," these appearances should be considered as accidental to hydrocephalus, just as suppuration, and tubercles of the lungs and the mesentery, are to pleurisy and peritonitis: they may follow the more chronic forms as a consequence, from the inflammation extending itself from the serous to the subjacent cellular membranes; or the inflammation of the serous membrane may be an accident to that of the cellular texture,—as we see in tubercles of the lungs, which go on a long time without producing much distress, but, if the inflammation extend to the pleura, hydrothorax is the immediate consequence. This is often seen: one lung will have only tubercles,—here is no lymphatic effusion into the chest; the other will have inflammation of the pleura, which may be traced to the tubercles, and here is hydrothorax on this side. But these are considered as combinations of disease in the chest, and they should be so considered in the brain. Dr. Cheyne repeatedly argues, as if it favoured his opinions, that hydrocephalus almost always is found to attack scrofulous children: we have just explained how it is that hydrocephalus so commonly thence results; that is, from the tubercles formed in the cellular texture of the brain at length exciting inflammation in the surrounding parts, which, extending to the serous membrane, the arachnoid, produces the symptoms during life, and the appearances after death, which are always found after inflammation of serous membranes, and after that of those membranes alone. But all these circumstances certainly militate against the doctrine of the gastric origin of the disease.

The only solid basis for nosography, is a knowledge of the texture that is affected, and the nature of the diseased action. Now, in the most simple form of *hydrocephalus* and of *phrenitis*, the affected texture is the arachnoid membrane, and the nature of the disease of it inflammation: this is shown by the circumstances attending the origin of the disease, by its symptoms and signs, and by the appearances after death.

It is not the dura mater that is affected in either disease: that never secretes such a fluid as is found in hydrocephalus, and the symptoms are not those of inflammation of a part of the fibrous texture: besides this, the arachnoid remains closely attached over the whole surface of the dura mater. The same remarks will equally apply to the pia mater: the fluid is contained in the sac of the arachnoid, and is not in contact with either of the former membranes; and this enclosure of the fluid shows that it cannot be the cellular texture of the brain that is affected. That texture may become affected, but it is only from the disease extending to it from its original seat; and disorganization of it is not found in the more simple and acute forms of the malady.

We, then, conclude, with Rush, that hydrocephalus is the phrenitis of children.

Though we thus differ from Dr. Cheyne respecting some points in the pathology of hydrocephalus, and as to its most frequent origin, we must at the same time admit, that it is sometimes a sympathetic affection of gastric disorder; and we cannot but admire his forcible picture of the history of the symptoms of the disease. We suspect that some local circumstances have influenced his observations; and we are the more inclined to do this, from the many well-marked cases that have lately been cured by blood-letting alone. A more free use of this remedy in many cases, and the resorting to it as the first measure, are, then, the only therapeutical precepts we would more forcibly inculcate than Dr. Cheyne has done in his general and particular directions for the treatment. We shall, therefore, notwithstanding the length to which this article has already extended, give a summary of his therapeutics in this disease.

After some general reflections on the varieties of the means proper for different cases, and especially on the importance of bearing in mind that inflammation of an organ, or other part, may exist, and require depletory measures for its removal, whilst the general system is in a state of debility, the author directs his remarks especially to the state of the alimentary canal. He says, in perhaps every instance, upon the first appearance of symptoms of hydrocephalus, it will be proper to use some active cathartic medicine; and, if the intestines be torpid, and continue to form vitiated secretions, it will be proper to pursue steadily for some time the purgative plan; and we ought to prescribe "the largest dose which can with safety be given of some powerfully cathartic combination, of which a mercurial ought to be an ingredient, two, three, or four, times a-day; and this to be continued for several days, or until healthy stools are procured. But," Dr. Cheyne continues, "not unfrequently the most active purgatives will be unavailing; which arises from an excitement of the mucous membrane, and perhaps in part from an inverted motion of the alimentary canal. As combined with nausea, it is always difficult to rectify this condition of the bowels; but, when the strength of the patient is such as to render the practice admissible, it will be remedied by general blood-letting." If the patient wince when the right hypochondrium is pressed, leeches ought to be applied to it, or the margin of the ribs cupped and scarified. After blood-letting, Dr. Cheyne says, "a drachm or two of magnesia saturated with lemon-juice, given every second or third hour, will often be more effectual than the most powerful purgatives;" and he adds, "in the cases in which hydrocephalus seems most remarkably to have its source in a disorder of the abdominal viscera, and in which the cure is to be effected by exciting those organs to free secre.ion, we are generally unable, after the first day or two, to effect that purpose by direct means." We have, under such circumstances, seen immediate and very remarkable good effects result from a warm-bath, when accompanied with the foregoing measures: the nausea has ceased, and evacuation from the bowels has speedily ensued. This measure seems, also, to have more extensive beneficial influence on the disease, by restoring the balance of vital action, or at least by removing determination to internal organs. The cathartics should, in general, be calomel with small doses of some common purgative of an active kind, as rhubarb, jalap, or scammony, with aloes: but, if there be a sickly smell of the breath, and fullness and uneasiness at the pit of the stomach, irritation of the mucous membrane of the intestines is denoted, which is sometimes best removed by local blood-letting and antimonials; we would add, too, the warm-bath. The following aphorism must be given in the author's own words:

"If the viscera of the abdomen, and particularly the liver, are in a state of high irritation, this irritation ought to be allayed before the stimulus which increases their secretions can be employed with advantage, or even with safety. The true practice is, in the first place, to reduce arterial action by venesection, or by topical bleeding and blistering; and then to restore the secerning function of the viscera, by means of calomel and other cathartics."

The foregoing remarks apply to nearly all cases of the disease: the other measures, especially the use of blood-letting, must be regulated by the constitutional disposition, the age, and strength, of the child, and the stage of the malady.

Vesicatories, after leeches, applied to the region of the liver, will frequently be necessary to prepare the way for the use of medicines to restore the proper functions of that organ.

After endeavouring to subdue the disorder of the bowels, the author says, there ought to be no delay in commencing a course of mercury: the efficacy of it, when resorted to in the manner here indicated, is acknowledged by every practitioner. It is desirable to produce some degree of salivation, that we may have evidence of its effects; but this will not be easily effected, if much excitement of any particular organ be present: this must be first subdued by the measures already inculcated.

Blisters are very powerful auxiliaries, especially after blood-letting, in removing the excitement both of the abdominal viscera and of the brain: cold applications to the head will also be advisable in some cases, with the same view. Remarkable benefit has been derived from the use of antimonial powder, and some of the milder attacks of the disease have been removed by this remedy alone. Opium is beneficial in the latter stages of the disease, especially when conjoined with mercury, by alleviating the pain and disposition to convulsions, and by producing quiet sleep. It may perhaps tend, too, to remove the remaining excitement.

Dr. Cheyne considers, in a particular manner, the use of digitalis in this disease, but he is not disposed to think favourably of its beneficial powers. We agree with him, as regards acute hydrocephalus; but we have witnessed some remarkable instances of its powerful agency, conjoined occasionally with purgatives, in the cure of chronic hydrocephalus. One case, in which the integuments were much raised above the anterior fontanel, accompanied by fluctuation, and where there was perfect paralysis of the lower limbs, in a child three years of age, was perfectly cured by this means. The child died four years afterwards from the measles.

Of course, though the effused fluid in this disease be chiefly situate over the hemispheres of the brain, the drawing-off the water by an operation is not likely to be often of much utility; since, as it has already been shown, the presence of this is not the most important circumstance: it is the diseased state of the brain causing this effusion that requires our attention; but it would appear, notwithstanding the unfavourable results which formerly attended that operation, that it should not be altogether abandoned in chronic hydrocephalus. It has lately been resorted to with success by Dr. Vose of Liverpool, and, as we have been informed, by Dr. Glover of the United States,—(a particular history of which case we expect to be able to give, very soon, in this Journal.) Dr. Rossi of Parma, a few years since, employed it in a case occurring after a fall, in a patient twelve years of age, where the parietal bones were separated. Six ounces of serum were then drawn off. Hippocrates seems to speak of it as a common operation, and in acute inflammation of the membranes of the brain, too; for, after having given a tolerably accurate description of hydrocephalus, (*ὕδωρ επι του εγκεφαλω*,) noticing the acute, darting, pains; rigors; dilated pupils; blindness; distension of the integuments of the cranium by the fluid, and the diaphanous appearance of the tumor; and spoken of the curative measures, he directs, if these are unsuccessful, a preliminary regimen; and then "let an incision be made in the head, just at the sinciput, and carried down to the brain, and the opening then closed, and healed in the ordinary way."*

* See Περὶ νουσων, β' § xv. See also § xvii. The whole of this book merits particular attention: the different varieties of inflammation of the contents of the cranium are distinguished in it with remarkable accuracy.

The legitimate object of this operation must, of course, be only to remove an effect of disease, that might prove fatal after that disease has disappeared.

We cannot enter into the consideration of the prophylactic measures: they will, indeed, be evident to the observant practitioner. This we may however state: Several facts have occurred to the knowledge of Dr. Cheyne, that seem ~~to~~ show the great efficacy of issues in preventing the disease, when there has been a family disposition to it, and even of removing the preliminary symptoms of it, when they have been manifested under the circumstances alluded to.

A very valuable collection of cases, with remarks, illustrate this treatise, from which the practitioner will derive a multitude of useful particular indications for the cure, as well as observations respecting the nature and progress, of the disease. Although we differ from the author in some points of pathology, we must not neglect to express the sense we entertain of the great value of this work. His doctrine respecting the origin of the disease is undoubtedly true in many cases; and very probably correct as far as his observations have extended; he has the merit of originality with respect to that doctrine: and, where it may not be strictly applicable to the pathology of this malady, it affords indications of the highest importance, which had not previously been discerned. His mode of treatment, too, has become almost generally adopted; only varied occasionally, from the indications of peculiar circumstances; and, as clinical histories, these essays are not excelled: they will bear with honour a comparison to any thing left us by Sydenham. But we have so often been obliged to remark the author's talent in this respect, that it is almost superfluous to point out the evidence of such a character in the present work.

---

*Medico-Chirurgical Transactions.* Vol. X. Part I.
London, 1819. Longman and Co.

[*Continued from page* 59.]

*Cases of Tumors within the Pelvis impeding Parturition; with Remarks.* By SAMUEL MERRIMAN, M.D. F.L.S. Physician-Accoucheur to the Middlesex Hospital, and to the Parochial Infirmary of St. George, Hanover-square; and Consulting Physician-Accoucheur to the Westminster General Dispenary.

THE tumors in these cases were, excepting in one instance, diseased ovaries. Dr. Merriman, in relating these, has collected several histories of a similar kind, detailed by other authors, and given a very useful summary of the results of the different modes of practice that were employed in the cases in which they occurred. He himself here furnishes accounts of five cases; but, from the useful practical inferences that may be deduced from them being so closely connected with a detail of numerous particular circumstances, which cannot be comprised in an abstract, we must refer the obstetric practitioner to the originals for the valuable information with which they will furnish him. However, as Dr. Merriman has himself drawn some general conclusions from them, we may transcribe these without the hazard of furnishing the reader with a false view of the practical indications his cases convey.

"I have been informed, that a very eminent teacher of midwifery used, in his Lectures, to assert, that 'the idea of treating these tumors by puncturing them, was very dangerous and highly improper;' and he taught that, on all occasions of this kind, the perforator should be early employed. But it may be doubted whether this opinion is tenable; for it has been proved that the force necessary to terminate the labour after the cranium has been perforated, has been so great as sometimes to cause the death of the patient, and sometimes to render her future life comfortless and distressful. In cases, therefore, where the tumor is found to intrench greatly upon the capacity of the pelvis, the perforator *alone* cannot be trusted to; neither does experience warrant the practice of turning and delivering by the feet.

"I know not that the Cæsarean operation has ever been applied to cases of this nature, and its constant fatality in this country would be a very great objection to employing that mode of giving relief. Yet it must be acknowledged, that if the Cæsarean operation had been performed upon Mrs. Daly at the time the puncture was made into the tumor, there would have been a great probability of preserving the child, which was then vigorous and active; and the consequences to herself could not have been more calamitous than resulted from her actual labour, conducted, as we believed, with the greatest caution and judgment. Had the tumor, in this case, been incapable of diminution by puncture, no other means of effecting delivery could have been used than the Cæsarean section; and consequently, under such circumstances, that operation would have been justifiable.

"Upon the whole, the evidence we at present possess, is most in favour of opening the tumors; for, of the nine women who recovered more or less perfectly, five appear to owe their safety to this operation; and, of the three children born alive, or supposed to be so, two were preserved by the same means. It may perhaps be necessary to ascertain, if possible, whether any advantage is likely to accrue from making an incision into the tumor from the vagina, rather than to puncture it through the rectum. The reasons by which we were influenced in choosing the latter were chiefly these: first, because the most depending part of the tumor could be reached from the rectum; and next, because we feared that an extensive laceration of the vagina might be produced by the stretching of the parts, when the child should come in contact with the incision or aperture.

"The following suggestion of Mr. Chevalier upon the subject of the after-management of the tumor, is too important to be withheld.

In a note which I received from him, respecting Mrs. Daly's case, he says, 'I submit to you how far it might be worth while to notice a circumstance which struck me as of some importance. You will recollect that, on the examination, I very easily, by my finger, dislodged the tumor from its situation between the rectum and the vagina, and returned the diseased ovarium into its place, before any dissection of the parts was attempted. Would it not in a similar case be right to attempt this, when the uterus is contracted after delivery, to prevent the tumor from fixing, by any subsequent inflammation, in that unnatural situation, where it might impede a future labour, more than it could do if loose in the abdomen, or adherent to the side?'"

*An Account of a Substance obtained from a diseased Ovarium; with some Remarks on diseased Secretions of an analogous Nature.* By John Bostock, M.D. F.R.S. and L.S.

This paper relates to the foregoing one, and can only be interesting in connexion with a particular history of the cases related. Dr. Bostock, however, has also indulged in some reflections on diseased secretions in general; but, as he has adduced them only as speculative notions, it would not be right to disfigure them by a partial account of them.

*On the Operation for Aneurism.* By George Norman, esq. of Bath. Communicated by Mr. Astley Cooper.

Mr. Norman's principal object in this Memoir is to adduce some observations in favour of the practice of applying but one ligature to the artery in the operation for aneurism, according to the mode of Hunter. Since this has lately become the most common, and indeed the general, practice in London, we shall not enter into any particular consideration of the cases related by Mr. Norman in this point of view; but there are one or two circumstances connected with them, on which we may make some remarks, that may be useful to some of our readers.

The first case given by Mr. Norman was one of inguinal aneurism. The patient's attention was first elicited to the part by his being suddenly seized with a severe pain in the upper part of the thigh, when he discovered a tumor "about the size of a pigeon's egg." The pain and swelling increased, and, according to the patient's account when brought to the hospital at Bath, in three days it had acquired the bulk then observed, which was about four inches from above downwards, and seven inches in width. This was at the end of nine days. He had been twice bled; and, the skin over the tumor being hot and red, the pulse 108, hard and full, that operation was repeated by direction of Mr. Norman, and cold lotions were applied to the tumor. Six days afterwards, a ligature was applied to the external iliac artery. Seven hours after the operation, sixteen ounces of blood were drawn from the arm to relieve flushing of the face, laborious breathing, &c.; and this was repeated on the third day. Cold lotions were applied to the tumor. He went on well until the 13th day, the tumor until this time gradually diminishing; but it again became somewhat increased in size, was hot and red, and pressure on it gave pain. *An evident pulsation could be felt in the tumor.* The author has not mentioned whether or not this had previously been observed. We notice this, because there are some circumstances connected with this *return* of pulsation in the aneurismal tumor, that are curious, and of much practical importance. It is probable, however, that, in Mr. Norman's case, there was continued pulsation from the time of the operation, because this in general happens in recent aneurisms. But, we allude to the recurrence of it in cases where there has been no pulsation in the tumor immediately after the operation, and when, too, the artery, for a little distance below it, has not pulsated for some time previously. This fact seems to have been first noticed by M. Larrey,* and he attributes it to the same causes which make the blood return from the extremities of an artery, and even the corresponding veins, on puncturing the trunk of the former, under certain states of the system; the local irritation causing an antiperistaltic motion of the vessels, which he says he has had the most decisive evidence of. This had been previously noticed by Bellini, Haller, Spallanzani, Soemmering, Barthez, Sabatier, and other equally accurate observers: this pulsation in the aneurismal sac, however, always subsides in a few days when anastomosing branches do not join the artery within a short distance below it. This will explain why secondary and fatal hemorrhage sometimes occurs when the Hunterian operation has been performed for an aneurism that has burst externally, and why in cases of aneurism arising from sudden rupture of the artery, especially in popliteal, extensive infiltration of the limb with blood will continue to take place after the operation has been performed. In both these cases, then, whenever it can be done, the old operation is preferable: that is, the tying the artery above and below the sac.

In Mr. Norman's case above mentioned, the inflammation of the integuments over the aneurismal sac was fortunately subdued by bleeding, cold lotions, and other appropriate measures; but, had suppuration taken place, it would probably have been followed by fatal consequences.

Perhaps, it may be possible to divert this antiperistaltic action

---

* In his Campagnes Chirurgicales, tom. iv. He has also recently noticed an instance in which it occurred after the Hunterian operation for aneurism. See *Bulletin de la Faculté de Medecine de Paris*, 1819. No. I. *in the Nouveau Journal de Medecine, tom.* iv.

of the cuticle, by producing severe irritation of the limb towards its extremities, by blisters and mustard sinapisms; whilst cold is applied to the region of the aneurism. It is not at all probable that the simple afflux of blood into the artery, at any considerable distance below the aneurism, could produce a fluctuating pulsation in this part; but that there must also be a determination of vascular action to the *irritated part*, (as it happens when the artery is punctured in the experiment of Bellini). The experiments of Dr. Jones, and of a multitude of other physiologists, sufficiently prove that, as soon as the action of an artery ceases, a coagulum begins to form throughout the whole portion of it thus affected.

The next case occurred under nearly analogous circumstances: the patient was, however, in a very bad state of health previously to the operation: "his countenance was exceedingly anxious, he was much emaciated, and his pulse was very quick and weak." He died twelve days after the operation, apparently from gangrene of the abdominal fascia, and inflammation of the peritoneum approaching the same state. The wound had sloughed too, but the process for obliteration of the artery and the cure of the aneurism, had been going on very well.

We shall transcribe Mr. Norman's remarks on the mode in which he performed the incision in the abdomen, in this case, as his reasoning is judicious.

"The operation was performed by making the incision in the direction of Poupart's ligament, as proposed by Mr. Astley Cooper, which I found to be a much more easy method of finding the external iliac artery, than the longitudinal incision practised by Mr. Abernethy, and which in the former case I had made use of.

"And it appears to me that the objection made to Mr. Cooper's mode of operating in cases where the tumor extends high up, is by no means well founded; for the lower part of the bag of the peritoneum, lying on the edge of Poupart's ligament, must in every case be exposed and detached, in order to get at the artery which lies behind the posterior part of that membrane; and this is most readily effected by an incision in the direction of Poupart's ligament, whilst two-thirds of the longitudinal incision are made on a part of the peritoneum which lines the abdominal muscles, and the lower portion only of the incision reaches that part of the membrane which is to be separated. The consequences of this are, that the peritoneum is in much greater danger of being wounded, and that the probability of a hernia forming after the cure is much increased by the extensive division of the oblique muscles."

Mr. Norman also relates the case of a boy, not quite fourteen years of age, who received a wound from a hay-fork in the upper and outer part of the left thigh. After having lost an immense quantity of blood, and an ineffectual attempt to discover the bleeding vessel by all the surgeons of the village near which he lived, he was bought to the hospital at Bath. We should have remarked, the hemorrhage had ceased for fifteen days, after having appeared at intervals for several days. But exactly a month after the accident, it burst out again, when the attempt to discover the wounded vessel above alluded to was made. It was the day after this that he was sent to Bath.

"On removing the tourniquet and bandage, the blood began to flow freely, but pressure on the artery in the groin commanded the hemorrhage. The opening in the outer part of the thigh was large enough to admit the whole hand. The wound made by the hay-fork was a little below the trochanter, but all trace of the direction in which the instrument had passed, was lost in the immense cavity formed among the muscles by the extravasated blood. The finger, when passed into the upper part of the cavity, reached the spot where my assistant compressed the artery in the groin.

"Under these circumstances, I was unwilling to hazard any attempt to find the wounded artery, more especially as the very reduced state the boy was in, rendered it certain that he could not survive any farther loss of blood, and as the passing of the finger to Poupart's ligament rendered it possible that the wound of the artery was high up. I therefore, whilst the assistant's thumb compressed the vessel, made an incision in the direction of the upper edge of Poupart's ligament, and passed a single ligature round the external iliac artery.

"After tying the ligature, I found that though the violence of the hemorrhage was restrained, yet that a larger quantity of blood continued to flow from the wound in the thigh than was consistent with the boy's safety.

"I therefore exposed the femoral artery two inches below Poupart's ligament; and, finding that when I held the artery between my finger and thumb, all bleeding ceased, I tied a single ligature round the artery."

The subsequent history of this interesting case is too long to be detailed: we can only state that sphacelation of the foot began to appear about the thirteenth day after the application of the ligatures, which extended up the leg; and, about a fortnight afterwards, the leg was amputated above the knee. The poor fellow finally recovered.

The other cases related by Mr. Norman do not present any circumstances of remarkable novelty or particular interest.

*On Urinary and other Morbid Concretion.* By William Henry, M.D. F.R.S. &c.

An account of this paper is given in the Historical Sketch of the Progress of Medicine.

